# Supplementary material for: Monocarboxylate Transporters 1 and 4 and Prognosis in Small Bowel Neuroendocrine Tumors
Source: Cancers (Basel). 2022 May 22;14(10):2552. doi: 10.3390/cancers14102552 (PMC9139933; doi:10.3390/cancers14102552)
Supplement: Supplementary file 1 [file cancers-14-02552-s001.zip › cancers-1689040-supplementary.pdf]

Supplementary material for article: Monocarboxylate Transporters 1 and 4 and Prognosis in Small Bowel Neuroendocrine Tumors

Authors: Niko Hiltunen [1,*], Jukka Rintala [1,2], Juha P Väyrynen [1], Jan Böhm [3], Tuomo J Karttunen [1], Heikki Huhta [1] and Olli Helminen [1,2]

1 Cancer and Translational Medicine Research Unit, Medical Research Center, University of Oulu and Oulu University Hospital, Oulu, Finland

2 Surgery Research Unit, Medical Research Center, University of Oulu and Oulu University Hospital, Oulu, Finland

3 Department of Pathology, Central Finland Central Hospital, Jyväskylä, Finland

* Correspondence: niko.hiltunen@oulu.fi

Supplementary material includes three Figures, where ROC curve analyses of MCT1 and MCT4 expression in primary small bowel neuroendocrine tumors, and MCT4 expression in small bowel neuroendocrine tumor lymph node metastases are presented.

Figure legends:

Supplementary Figure S1: ROC curve analysis of disease-specific survival related to MCT1 intensity and MCT1 percentage in primary tumors showing no prognostic significance with any cut-off value.

Supplementary Figure S2: ROC curve analysis of disease-specific survival related to MCT4 intensity, MCT4 percentage and MCT4 stroma in primary tumors proposing optimal cut-off (furthest point from reference line) values of 2 in staining intensity and >95 in percentage of positive cells. MCT4 stroma showed no prognostic significance with any cut-off value.

Supplementary Figure S3: ROC curve analysis of disease-specific survival related to MCT4 intensity, MCT4 percentage and MCT4 stroma in lymph node metastases proposing optimal cut-off (furthest point from reference line) values of 2.5 in staining intensity and >85 in percentage of positive cells. MCT4 stroma crossed the reference line and showed no prognostic significance.

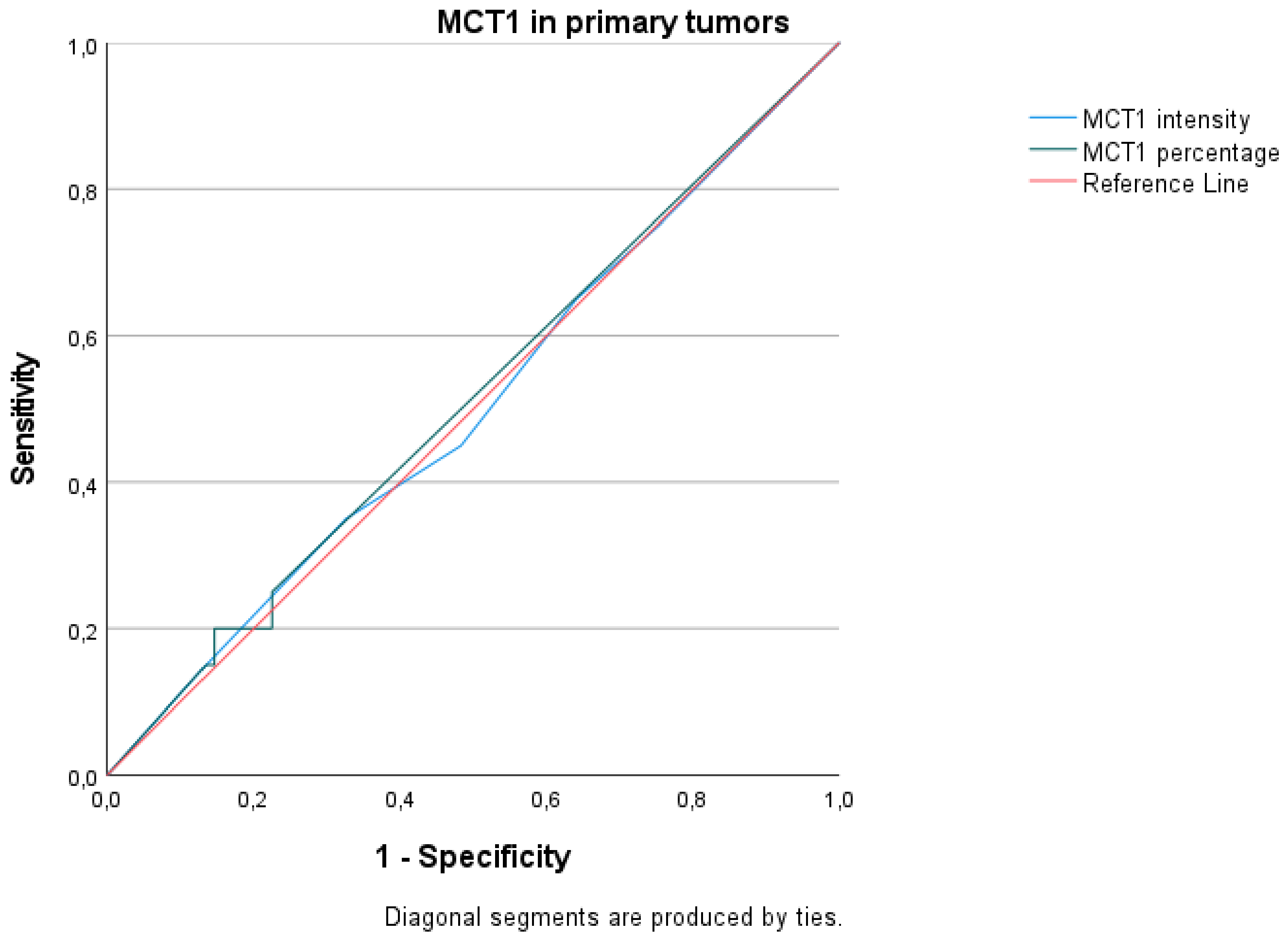

Supplementary Figure S1.

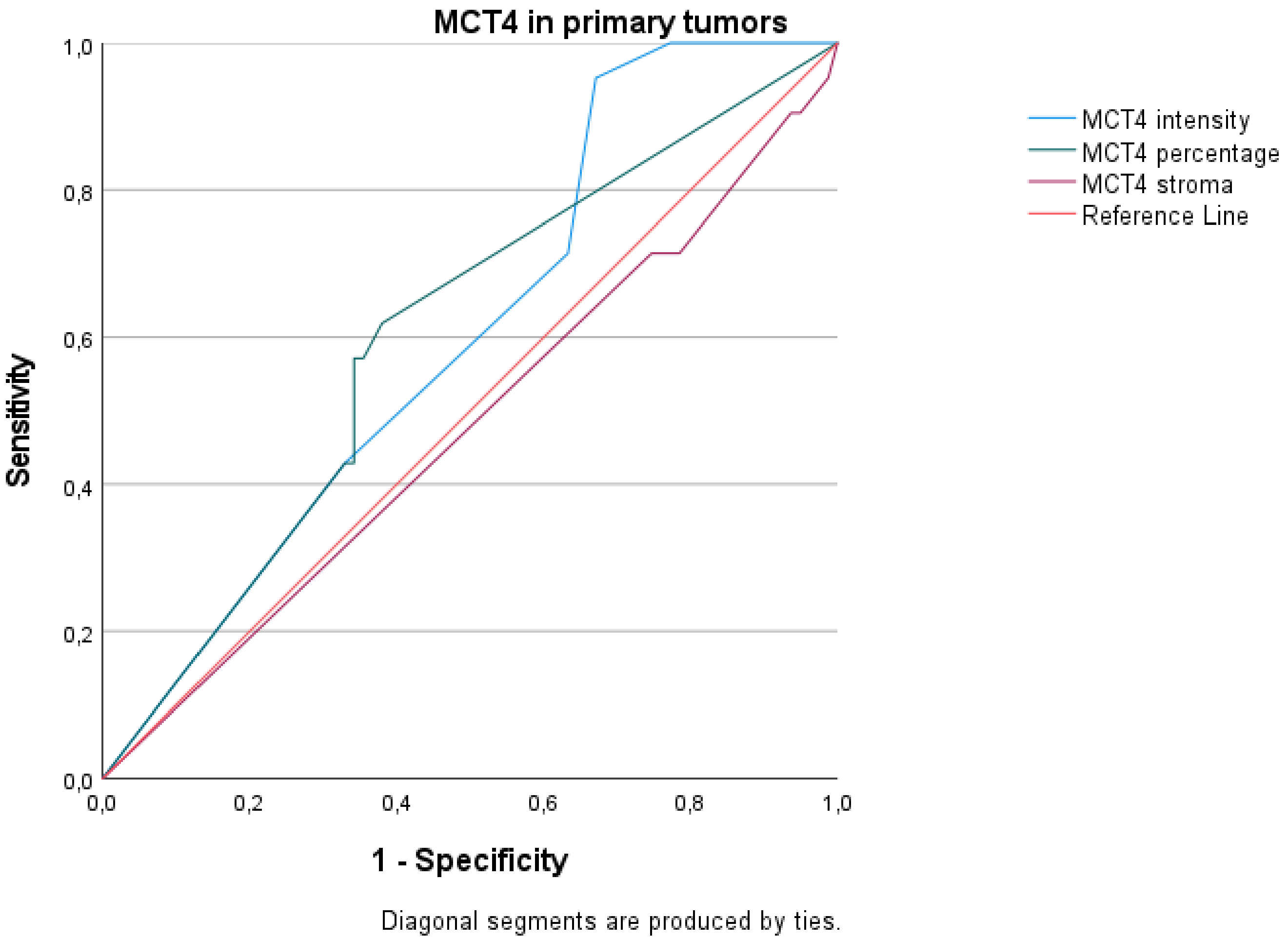

Supplementary Figure S2.

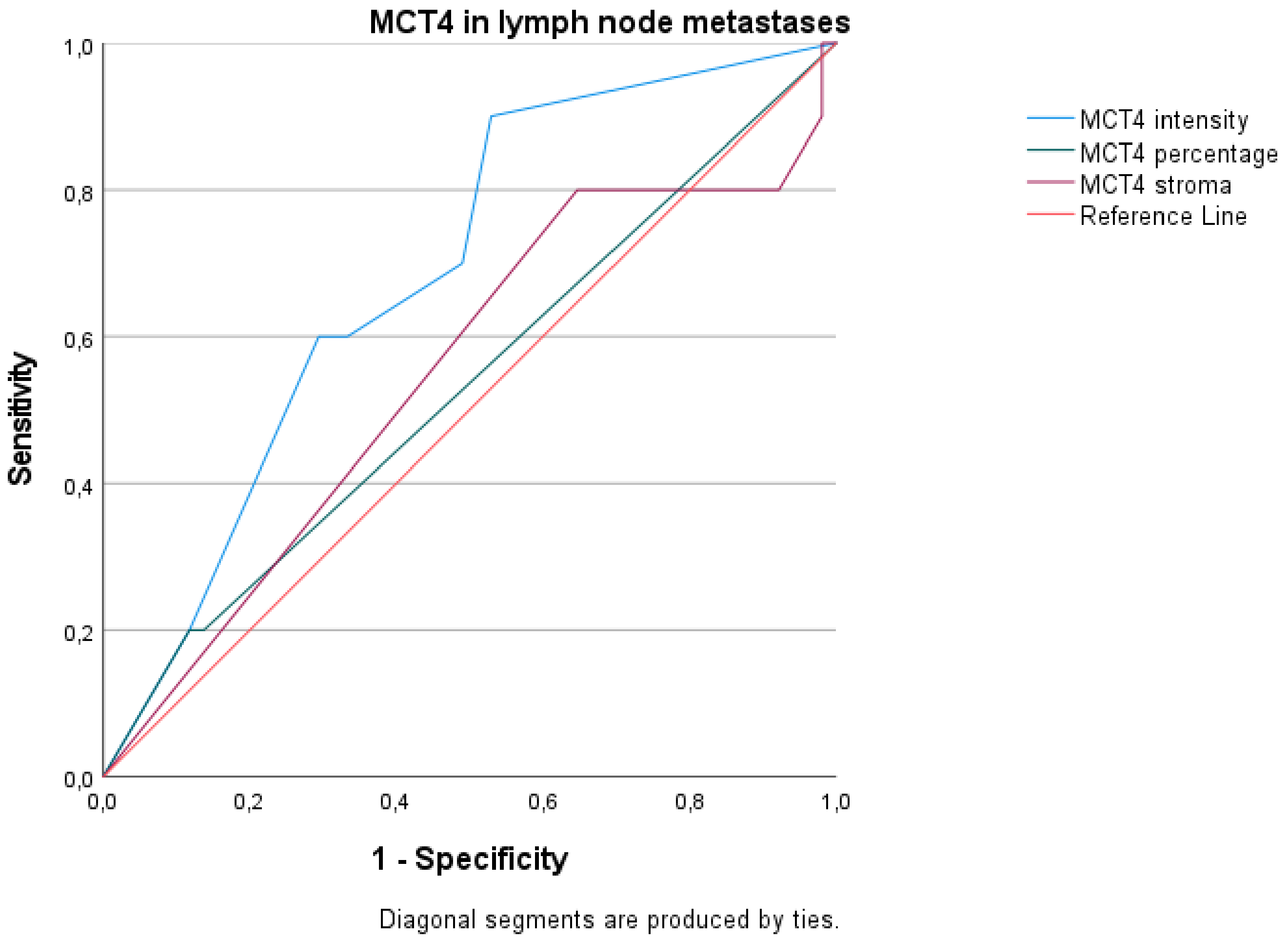

Supplementary Figure S3.
